# Elevated ASGR1 as a Potential Diagnostic Biomarker for Coronary Artery Disease and Predictor of Adverse Outcomes in Hypertensive Patients

**DOI:** 10.1096/fj.202504652RR

**Published:** 2026-04-28

**Authors:** Ying Liu, Xinyu Zhou, Peng Hao, Ying Guo, Ning Wang, Kuibao Li, Hongjie Chi, Hong Li, Xiaoyan Yang, Jing Li, Jiuchang Zhong, Lin Zhao, Ying Dong

**Affiliations:** ^1^ Heart Center and Beijing Key Laboratory of Hypertension, Beijing Chaoyang Hospital, Capital Medical University Beijing China; ^2^ Department of Cardiology Beijing Chaoyang Hospital, Capital Medical University Beijing China; ^3^ Department of Geriatrics Beijing Tongren Hospital, Capital Medical University Beijing China; ^4^ Medical Research Center, Beijing Chaoyang Hospital, Capital Medical University Beijing China

**Keywords:** ASGR1, coronary artery disease, hypertension, myocardial infarction, rehospitalization

## Abstract

Asialoglycoprotein receptor 1 (ASGR1) is associated with lipid metabolism and coronary artery disease (CAD) risk, but its expression patterns, diagnostic performance, and prognostic significance in hypertensive patients with CAD remain unelucidated. This single‐center study enrolled 345 hypertensive patients between 2022 and 2025 (59 with hypertension alone, 286 with hypertension and CAD). Plasma ASGR1 levels were measured by enzyme‐linked immunosorbent assay. Receiver operating characteristic (ROC) curves, Spearman correlation, and Cox proportional hazards modeling were performed to assess the diagnostic efficacy of ASGR1 in CAD and its prognostic value for all‐cause rehospitalization. Plasma ASGR1 levels were significantly higher in hypertensive patients with CAD than in those with hypertension alone (*p* < 0.001) and higher ASGR1 expression is accompanied by more severe coronary lesions and adverse clinical phenotypes. Inflammatory markers, liver injury biomarkers and cardiac injury biomarkers were positively correlated with ASGR1, whereas high‐density lipoprotein cholesterol (HDL‐C) was negatively correlated. ASGR1 showed excellent diagnostic ability for CAD in hypertensive patients with area under the curve of 0.937 (95% CI: 0.906–0.960). Multivariate analysis showed that each 1‐unit increase in ASGR1 was associated with a 37% higher risk of CAD (odds ratio 1.37, 95% CI: 1.24–1.52, *p* < 0.001). Longitudinally, elevated baseline ASGR1 was independently associated with an increased risk of all‐cause rehospitalization (adjusted hazard ratio 1.97, 95% CI: 1.16–3.35, *p* = 0.012). These findings support ASGR1 may serve as a diagnostic biomarker and prognostic indicator for hypertensive patients with CAD.

## Graphical abstract

Asialoglycoprotein receptor 1 (ASGR1) is linked to lipid metabolism and CAD, but its role in hypertensive CAD patients is unclear. This single‐center study included 345 hypertensive patients (59 non‐CAD, 286 CAD). Plasma ASGR1 was higher in CAD patients, correlated with multiple markers and HDL‐C, had high diagnostic value (better than traditional biomarkers), and predicted all‐cause rehospitalization risk, serving as a promising diagnostic and prognostic biomarker for hypertensive CAD.

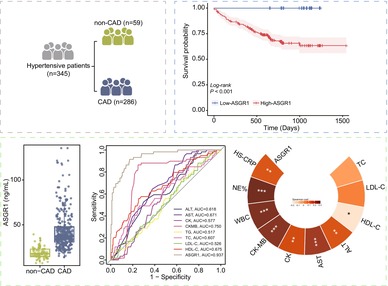

## 1 Introduction

Coronary artery disease (CAD), a chronic inflammation‐driven disorder, constitutes substantially to global morbidity and mortality [1]. In China, CAD affects an estimated 11.39 million adults, with rising mortality particularly in rural regions [2]. Currently, high‐sensitivity cardiac troponins represent the most widely used and recommended biomarkers for acute coronary syndromes (ACS) [3]. Other conventional markers, including N‐terminal pro‐B‐type natriuretic peptide, creatine kinase‐MB (CK‐MB), high‐sensitivity C‐reactive protein (hs‐CRP), and Interleukin‐6 (IL‐6), are also commonly applied. However, these biomarkers have several limitations, such as low specificity for ACS‐related atherosclerosis and inflammation, limited ability to reflect coronary lesion severity, and potential interference from gender, age, obesity, and other non‐cardiac conditions [4]. Thus, novel stable and specific biomarkers are urgently needed to improve early screening, risk stratification, severity elevation, and prognostic assessment of CAD.

Hypertension is a well‐established major risk factor for CAD [5, 6]. Given their shared multifactorial pathogenesis involving genetic, environmental, and lifestyle‐related factors, their combination exerts synergistic adverse effects on cardiovascular health [7, 8, 9]. Effective initial screening and preventive strategies are therefore critical for this high‐risk population.

Asialoglycoprotein receptor 1 (ASGR1) belongs to the lectin family, a large group of proteins that bind carbohydrate structures via non‐enzymatic mechanisms [10]. Existing literature demonstrates that human heterozygote carriers of *ASGR1* deletions exhibit 34% lower CAD risk and a 10%–14% reduction of non‐high‐density lipoprotein cholesterol [11, 12, 13]. Preclinical studies indicated that ASGR1 deficiency reduces atherosclerotic lesions and enhances cholesterol efflux, supporting a protective role against cardiovascular disease [14]. In addition, serum ASGR1 levels were significantly higher in CAD patients compared with non‐CAD controls and remained independently associated with CAD risk after adjusting for confounding variables [15]. Beyond its established role in lipid metabolism and atherosclerotic CAD, emerging evidence suggests a potential interplay between ASGR1 and the pathophysiological processes underlying hypertension. Hypertension is characterized not only by elevated blood pressure but also by endothelial dysfunction, vascular remodeling, and a pro‐inflammatory state [16]. ASGR1 acts as a key receptor involved in inflammatory and metabolic regulation [17], supporting its potential involvement in the pathophysiological milieu of hypertension. However, prior population‐based studies have been exclusively cross‐sectional studies, limiting the ability to establish temporal relationships between ASGR1 and CAD pathogenesis. Additionally, ASGR1 expression levels in hypertension complicated by CAD remain uninvestigated.

Therefore, this study aims to investigate plasma ASGR1 levels in patients with hypertension complicated by CAD. Furthermore, through longitudinal follow‐up of these patients, we sought to evaluate whether baseline ASGR1 expression could predict rehospitalization rate. Our findings may provide novel insights into the early diagnosis, clinical management, and prognosis of hypertension‐associated CAD.

## 2 Methods

### 2.1 Study Population

In this single‐center, cross‐sectional study, consecutive patients were enrolled at Beijing Chaoyang Hospital Affiliated with Capital Medical University from June 2022 to January 2025. Consecutive adult patients with a diagnosis of hypertension, with or without CAD, were screened for eligibility. This study was approved by the Research Ethics Committee of Beijing Chaoyang Hospital Affiliated to Capital Medical University (No. 2022‐ke‐483) and was conducted in accordance with the principles of the Declaration of Helsinki. Written informed consent was obtained from all participants prior to data collection. Exclusion criteria included secondary hypertension, severe heart valvular disease, cardiomyopathy, severe hepatic and renal insufficiency, autoimmune disease, heart failure, thyroid dysfunction, pregnancy, complicated acute infection, acute cerebrovascular disease, active malignant tumor, or an estimated life expectancy of less than 2 years.

### 2.2 Definition

Hypertension was defined in accordance with the 2010 Chinese guidelines for the management of hypertension as systolic blood pressure (SBP) ≥ 140 mmHg, and/or diastolic blood pressure (DBP) ≥ 90 mmHg, or current use of antihypertensive medication within the previous 2 weeks [18]. In addition, CAD was diagnosed based on typical angina pectoris (oppressive, dull or constricting discomfort in the retrosternal or precordial region, usually lasting 3–5 min, and can be rapidly relieved by sublingual nitroglycerin), relevant electrocardiographic abnormalities, and invasive coronary angiography showing ≥ 50% luminal stenosis in at least one major epicardial coronary artery [15]. According to the 2024 European Society of Cardiology (ESC) guidelines, chronic coronary syndrome (CCS) refers to a spectrum of clinical disorders caused by structural and/or functional abnormalities of coronary atherosclerosis and/or microvascular dysfunction [19, 20]. Furthermore, in accordance with ESC guidelines [21] and ACC/AHA guidelines [22], ACS was defined as an acute clinical syndrome by acute, transient or persistent myocardial ischemia and hypoxia resulting from the rupture or erosion of coronary atherosclerotic plaques, followed by thrombosis formation. ACS is classified into three subtypes, including ST‐segment elevation myocardial infarction (STEMI), non‐ST‐segment elevation myocardial infarction (NSTEMI), and unstable angina (UA) [21].

### 2.3 Study Group Classification

A total of 345 patients were included, comprising 59 with hypertension alone (non‐CAD group) and 286 with hypertension and concomitant CAD (CAD group). Among patients with hypertension and CAD, further stratification was performed according to the extent of coronary artery involvement based on coronary angiography (CAG) findings, classifying patients into those with involvement of two or fewer coronary vessels (CAG ≤ 2, *n* = 110) and those with involvement of more than two vessels (CAG > 2, *n* = 176). In addition, patients with hypertension and CAD were also classified according to clinical presentation into CCS (*n* = 73) and ACS (*n* = 213) following ESC guidelines.

### 2.4 Data Collection

Demographic characteristics, medical history, physical examination findings, laboratory results, and treatment records were extracted from the hospital's electronic medical record system. We also documented use of antihypertensive medications and the most frequently employed agents included angiotensin‐converting enzyme inhibitors/angiotensin II receptor blockers, beta‐blockers, and calcium channel blockers. Blood pressure, smoking status, alcohol consumption, and body mass index (BMI, calculated as weight in kilograms divided by height in meters squared) were recorded during routine clinical care and subsequently retrieved from the electronic records. Patients were instructed to fast for at least 8 h before blood sampling. Fasting venous blood samples were collected from the antecubital vein by nursing staff using standard aseptic techniques. Whole blood was used for routine blood tests, including white blood count (WBC) and neutrophil percentage (NE%). Serum levels of fasting blood glucose (FBG), lipid profiles, alanine aminotransferase (ALT), aspartate aminotransferase (AST), creatine kinase (CK), CK‐MB, and hs‐CRP were determined using an automated biochemical analyzer (AU5800, Beckman Coulter, USA) [23].

### 2.5 ASGR1 Enzyme Immunoassay

Plasma was separated by centrifugation at 1500 g for 10 min at 4°C and stored at −20°C until analysis. Plasma levels of ASGR1 were measured using a commercially available sandwich enzyme‐linked immunosorbent assay (ELISA) kit (CSB‐E09130h, Shanghai Shunyuan Biotechnology Co. Ltd., China), following the manufacturer's instructions. Optical density was read at a wavelength of 450 nm using a Thermo Scientific Multiscan GO ELISA reader (Finland). The intra‐assay coefficient of variation was maintained below 10%.

### 2.6 Establishment of Myocardial Infarction Model

Eight‐week‐old male C57BL/6 mice (weighting 22–25 g, *n* = 10, Charles River Laboratories, Beijing, China) were housed under standard conditions (12:12‐h light/dark cycle, 25°C, 45% humidity) with ad libitum access to water and standard rodent chow [24]. Animals were randomly assigned to a sham‐operated group (*n* = 5) or a myocardial infarction (MI) group (*n* = 5).

For MI induction, mice were anesthetized with 2% isoflurane (HZB1682, Huahai Haiwei (Beijing) Gene Technology Co., Beijing, China) and maintained on a heating pad at 37°C [25]. A left thoracotomy was performed at the fourth intercostal space, and the left anterior descending coronary artery was permanently ligated using 7‐0 nylon sutures. Successful occlusion was confirmed by the appearance of myocardial blanching distal to the ligation site. Sham‐operated mice underwent an identical surgical procedure, except for left anterior descending ligation. Following surgery, the thoracic cavity was closed, and animals were allowed to recover under thermal support [26].

### 2.7 Echocardiographic Assessment of Cardiac Function for Humans and Mice

Transthoracic echocardiography was performed to assess cardiac structure and function in both humans and mice, with species‐specific adjustments to equipment, protocols, and protocols to ensure accuracy and relevance. For human examinations, all procedures were conducted by two experienced echocardiographers using a commercial ultrasound system (EPIQ 7c, Philips Electronics, The Netherlands) equipped with a 3.5/2.5 MHz transducer. Patients were examined in the left lateral decubitus position to optimize acoustic windows and image quality. Measurements were obtained in accordance with the recommendations of the American Society of Echocardiography [27]. M‐mode echocardiography was used to assess the following parameters: left ventricular end‐diastolic diameter (LVEDd), left ventricular end‐systolic diameter (LVEDs), interventricular septal thickness (IVST), left ventricular posterior wall thickness (LVPW), and left atrial diameter (LAD). LVEDd and LVEDs were measured as the distance between the endocardial surfaces of the interventricular septum and the left ventricular posterior wall at the time of maximal and minimal left ventricular chamber dimensions, respectively. The Teichholz method is a commonly used echocardiographic method for estimating left ventricular ejection fraction (LVEF%) based on linear dimensions measured from parasternal long‐axis views [28]. Left ventricular end‐diastolic volume (LVEDV), left ventricular end‐systolic volume (LVESV), and LVEF% were subsequently calculated: LVEDV = 7.0 × LVEDd^3^/(2.4 + LVEDd), LVESV = 7.0 × LVEDs^3^/(2.4 + LVEDs), LVEF% = (LVEDV–LVESV)/LVEDV × 100% [29]. Additionally, left ventricular mass (LVM) was estimated using the formula [30]: LVM (g) = 0.8 × [1.04 × (LVEDd + LVPW + IVST)^3^—LVEDd^3^] + 0.6.

For mice, transthoracic echocardiography was performed 8 weeks after MI or sham surgery using the VisualSonics Vevo 770 High‐Resolution In Vivo Imaging System (VisualSonics, Canda) fitted with a 30 MHz transducer (RMV‐707B; VisualSonics, Canda). Mice were placed in the supine position on a heated platform during imaging. To minimize cardiac depression and ensure stable physiological conditions, mice were anesthetized with 3% isoflurane for induction and maintained on 1.5% isoflurane in oxygen during the procedure. Body temperature was kept at 37°C using a heated platform, and respiratory rate was continuously monitored. The anterior chest of each mouse was shaved with depilatory cream to optimize the acoustic window. Echocardiographic parameters evaluated for mice included LVEDd, LVEDs, LVEF% and fractional shortening (FS%) (FS% = (LVEDd^3^−LVEDs^3^)/LVEDd^3^ × 100%).

### 2.8 Masson's Trichrome Staining

Myocardial collagen deposition was evaluated by Masson's trichrome staining. Briefly, cardiac tissue was fixed with 4% paraformaldehyde, embedded in paraffin, and microtome sliced into 5 μm sections. Sections were deparaffinized in xylene and rehydrated through a graded series of ethanol to distilled water. Nuclei were stained with Harris hematoxylin, differentiated in 1% acid alcohol, and blued under running water. Sections were then stained with ponceau fuchsin, differentiated with 1% phosphomolybdic acid, and counterstained with aniline blue (G1006, Servicebio Co. Ltd., Wuhan, China). The collagen content was measured using Image J software (National Institute of Health, Bethesda, MD, USA). The collagen volume fraction (CVF)% = collagen area/tissue total area × 100%.

### 2.9 Wheat Germ Agglutinin (WGA) Staining

To analyze the cross‐sectional area of cardiomyocytes, myocardial tissues were stained with WGA staining. Briefly, myocardial tissue sections were fixed in 4% paraformaldehyde for 30 min at room temperature. Following PBS washing, permeabilization was performed using 0.1% Triton X‐100 in PBS for 10 min. Sections were then blocked with 5% bovine serum albumin (BSA) for 30 min to minimize nonspecific binding. Alexa Fluor 488‐conjugated wheat germ agglutinin (G1730, Servicebio Co. Ltd., Wuhan, China) was diluted at 1:200 in blocking buffer and applied for 1 h at room temperature in the dark to prevent fluorescence quenching. After three PBS washes, nuclei were counterstained with 4′,6‐diamidino‐2‐phenylindole (DAPI, 1 μg/mL, G1012, Servicebio Co. Ltd., Wuhan, China) for 5 min. Stained sections were scanned using a Zeiss LSM 880 confocal microscope (Carl Zeiss Microscopy GmbH, Jena, Germany). Cardiomyocyte cross‐sectional area was quantified in Image‐Pro Plus (Media Cybernetics Inc., Rockville, MD, USA) by manually tracing WGA‐labeled sarcolemma.

### 2.10 RNA Extraction and Quantitative Real‐Time PCR (qRT‐PCR)

Total RNA of cardiac tissue was extracted using TRIzol reagent (Thermo Fisher Scientific) according to the manufacturer's instructions. After assessing the concentration and purity of the extracted RNA using NanoDrop 2000 spectrophotometer (Thermo Fisher Scientific), a total of 1 μg of RNA was reverse‐transcribed into cDNA using the GenePharma kit following the standard protocol. Subsequently, relative mRNA levels were quantified using qPCR quantitation kit (GenePharma), with GAPDH serving as the endogenous control. The relative expression levels were calculated via the 2^−ΔΔ^CT method. The specific primer sequences synthesized by Sangon Biotech were shown Table S1.

### 2.11 Statistical Analysis

Continuous variables are presented as mean ± standard deviation (SD) or median (Q1, Q3) and compared using Student's *t* test or the Mann–Whitney *U* test, as appropriate. Categorical variables are expressed as numbers (%) and compared using Chi‐square test. Spearman's rank correlation analysis was conducted to assess the associations between ASGR1 expression and three categories of indicators: inflammatory cytokine levels (including IL‐1β, IL‐6, and IL‐10), pathological indicators, and cardiac ultrasound parameters. To further evaluate the predictive capacity of ASGR1 for CAD, receiver operating characteristic (ROC) curve analyses were performed comparing ASGR1 with established risk biomarkers, including ALT, AST, CK, CK‐MB, TG, TC, HDL‐C, and LDL‐C. The discriminative performance was quantified by calculating the area under the ROC curve (AUC) for each marker, with statistical comparison of AUC values performed using DeLong's test (MedCalc software, version 11.4.2.0) [31].

In the cross‐sectional analysis, variables that exhibited statistically significant associations (*p* < 0.05) in the univariate analyses were retained for inclusion in subsequently multivariate modeling to evaluate the risk of CAD among patients with hypertension. Additionally, in the longitudinal analysis, restricted cubic spline (RCS) modeling was employed to evaluate both the overall and potential nonlinear relationship between continuous ASGR1 and all‐cause rehospitalization. The reference value was set at the optimal cut‐off point identified for ASGR1. Subsequently, according to the categorized ASGR1 based on the optimal cut‐off point (Low‐ASGR1: ASGR1 ≤ 17.945 ng/mL; High‐ASGR1: ASGR1 > 17.945 ng/mL), Kaplan–Meier curves were plotted to evaluate survival differences. Initially, univariate Cox regression analysis was conducted to evaluate the relationship between individual predictor variables and occurrence of rehospitalization. Subsequently, only variables with *p* < 0.05 in univariate analysis were included in the multivariate Cox proportional hazards models to investigate the association between categorized ASGR1 expression levels and the incidence of all‐cause rehospitalization. In these models, the low‐ASGR1 group—defined based on an optimal cut‐off value—served as the reference category. Covariates that demonstrated statistically significant associations (*p* < 0.05) in the univariate analyses were included in the multivariate models for adjustment. Results are presented as hazard ratios (HRs) with corresponding 95% confidence intervals (CIs).

Statistical analysis was performed using Graphpad Prism software version 9.5.1 (GraphPad Software, San Diego, CA, USA), with SAS 9.4 (SAS Institute, Cary, NC, USA) and with R version 4.1.3 (R Foundation for Statistical Computing, Vienna, Austria).

## 3 Results

### 3.1 Baseline Characteristics

The baseline demographic, clinical, and biochemical characteristics of the study population are summarized in Table 1. The population encompassed 345 participants with a mean age of 66.6 years, of whom 69.6% were male. Notable elevations were observed in several biochemical indices, such as ALT, AST, CK‐MB, hs‐CRP, WBC and NE%, and echocardiographic parameters derived from ultrasound assessments (e.g., IVST, LVPW, and LVM) among hypertensive patients comorbid with CAD. Also, a higher proportion of current smokers was observed in hypertensive patients with CAD compared to those with hypertension alone. Conversely, the hypertensive CAD group exhibited lower levels of HDL‐C and reduced LVEF%.

**TABLE 1:** Baseline characteristics of the study participants.

Variable	Total	Non‐CAD	CAD	*p*
No.	345	59	286	
Age (year)	66.6 ± 8.5	68.1 ± 7.0	66.3 ± 8.7	0.101
Sex (male%)	240 (69.6)	39 (66.1)	201 (70.3)	0.525
BMI (kg/m^2^)	25.7 ± 3.5	26.1 ± 3.2	25.6 ± 3.5	0.294
SBP (mmHg)	134.0 ± 18.0	134.9 ± 15.0	133.8 ± 18.6	0.643
DBP (mmHg)	75.7 ± 10.7	75.2 ± 10.4	75.8 ± 10.8	0.708
Antihypertensive medication	274 (79.4)	63 (88.7)	223 (81.4)	0.143
Smoking	107 (31.0)	10 (17.0)	97 (33.9)	0.010
Drinking	88 (25.5)	13 (22.0)	75 (26.2)	0.501
T2DM	180 (52.2)	33 (55.9)	147 (51.4)	0.526
FBG (mmol/L)	6.43 ± 2.75	6.26 ± 1.82	6.47 ± 2.91	0.459
TG (mmol/L)	1.18 (0.84–1.77)	1.21 (0.90–1.71)	1.15 (0.84–1.77)	0.673
TC (mmol/L)	3.91 ± 1.05	4.25 ± 1.14	3.84 ± 1.02	0.006
HDL‐C (mmol/L)	1.05 ± 0.37	1.27 ± 0.55	1.01 ± 0.30	< 0.001
LDL‐C (mmol/L)	2.41 ± 0.97	2.47 ± 0.90	2.40 ± 0.99	0.641
ALT, U/L	21.0 (15.0–31.0)	16.0 (13.0–25.0)	21.5 (15.0–33.0)	0.004
AST, U/L	22.0 (18.0–34.0)	19.0 (17.0–25.0)	23.0 (18.0–42.0)	< 0.001
CK, U/L	99.0 (65.0–195.0)	84.0 (63.0–124.0)	105.5 (65.0–250.0)	0.063
CK‐MB, ng/mL	50.0 (11.0–179.0)	11.0 (9.0–14.0)	70.0 (20.0–220.0)	< 0.001
Hs‐CRP, mg/L	1.50 (0.50–5.40)	0.80 (0.40–2.30)	1.60 (0.50–6.50)	0.005
WBC, ×109/L	7.40 (5.90–9.40)	6.10 (5.20–7.40)	7.90 (6.10–9.80)	< 0.001
NE%	67.34 ± 12.72	60.95 ± 9.42	68.66 ± 12.92	< 0.001
LVEDd, cm	4.76 ± 0.62	4.84 ± 0.75	4.75 ± 0.59	0.400
LVEDs, cm	3.13 ± 0.58	3.20 ± 0.46	3.11 ± 0.60	0.237
IVST, cm	1.03 ± 0.21	0.87 ± 0.16	1.06 ± 0.20	< 0.001
LVPW, cm	0.98 ± 0.17	0.88 ± 0.15	1.00 ± 0.17	< 0.001
LAD, cm	3.78 ± 0.54	3.69 ± 0.47	3.81 ± 0.56	0.134
LVEF, %	61.03 ± 9.34	63.93 ± 4.98	60.44 ± 9.92	< 0.001
LVM, g	165.5 (142.5–196.9)	145.2 (120.5–171.7)	173.4 (148.2–200.5)	< 0.001

Abbreviations: ALT, alanine aminotransferase; AST, aspartate aminotransferase; BMI, body mass index; CAD, coronary artery disease; CK, creatine kinase; CK‐MB, creatine kinase‐MB; DBP, diastolic blood pressure; FBG, fasting blood glucose; HDL‐C, high‐density lipoprotein cholesterol; Hs‐CRP, high sensitivity C‐reactive protein; IVST, interventricular septal thickness; LAD, left atrial diameter; LDL‐C, low‐density lipoprotein cholesterol; LVEDd, left ventricular end‐diastolic diameter; LVEDs, left ventricular end‐systolic diameter; LVEF, left ventricular ejection fraction; LVM, left ventricular mass; LVPW, left ventricular posterior wall thickness; NE%, neutrophilic granulocyte %; No, number; SBP, systolic blood pressure; T2DM, type 2 diabetes mellitus; TC, total cholesterol; TG, triglyceride; WBC, white blood count.

### 3.2 Plasma ASGR1 Levels in Hypertensive Patients With vs. Without CAD

**FIGURE 1:**
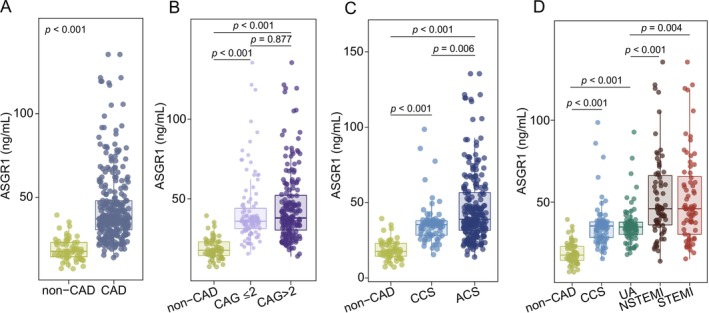
Comparative analysis of plasma ASGR1 levels across clinical subgroups in hypertensive with or without CAD. (A) Plasma ASGR1 concentrations in hypertensive with CAD (CAD, *n* = 286) or without CAD (non‐CAD, *n* = 59; Mann–Whitney *U* test, *p* < 0.001). (B) Plasma ASGR1 expression in hypertensive cohorts cross‐stratified by CAD phenotype (non‐CAD, *n* = 59) and angiographic vessel involvement (CAG ≤ 2, *n* = 110; CAG > 2, *n* = 176, non‐CAD vs. CAG ≤ 2, *p* < 0.001; non‐CAD vs. CAG > 2, *p* < 0.001; CAG ≤ 2 vs. CAG > 2, *p* = 0.877). (C) ASGR1 levels in hypertensive patients stratified by coronary syndrome phenotype: Isolated hypertension (non‐CAD, *n* = 59), CCS (*n* = 73), and ACS (*n* = 213) (non‐CAD vs. CCS, *p* < 0.001; non‐CAD vs. ACS, *p* < 0.001; CCS vs. ACS, *p* = 0.006). (D) Analysis of plasma ASGR1 levels in hypertensive patients stratified by CAD severity (non‐CAD, *n* = 59; CCS, *n* = 73; UA, *n* = 75; NSTEMI, *n* = 70; STEMI, *n* = 68) (non‐CAD vs. CCS, *p* < 0.001; non‐CAD vs. UA, *p* < 0.001; UA vs. NSTEMI, *p* < 0.001; UA vs. STEMI, *p* = 0.004). B‐D, Kruskal‐Wallis *H* test with Dunn's multiple comparisons test. ASGR1, asialoglycoprotein receptor 1; CAD, coronary artery disease; CAG, coronary angiography; CCS, chronic coronary syndrome; ACS, acute coronary syndrome; UA, unstable angina; NSTEMI, non‐ST elevation myocardial infarction; STEMI, ST‐elevation myocardial infarction.

Plasma ASGR1 levels were markedly elevated in hypertensive patients with CAD (*n* = 286) compared to CAD‐free hypertensive control (*n* = 59) [median (IQR): 37.16 (30.62, 48.03) vs. 17.69 (14.40, 23.16) ng/mL, *p* < 0.001] (Figure 1A). Compared with the non‐CAD group, plasma ASGR1 levels were increased by 2.10‐fold in CAD patients. Furthermore, we investigated the relationship between ASGR1 levels and the severity of CAD among hypertensive patients. As shown in Figure 1B, ASGR1 expression in hypertensive patients without CAD was significantly lower than in those with ≤ 2 and > 2 coronary artery branch involvement (assessed by CAG). Notably, although the CAG‐defined > 2 vessels group exhibited a slightly upward trend in ASGR1 expression compared to the ≤ 2 vessels group, no statistically significant difference was detected between the two groups (*p* = 0.877). Figure 1C demonstrated a stepwise increase in ASGR1 levels among hypertensive patients stratified into non‐CAD, CCS and ACS, with a 2.20‐fold increase in ACS patients relative to the non‐CAD group. Subgroup analysis in Figure 1D further revealed that within the ACS cohort, NSTEMI and STEMI patients had the highest ASGR1 levels, both of which were markedly elevated compared to UA patients (*p* < 0.001 and *p* = 0.004, respectively). In particular, STEMI patients showed a 2.60‐fold increase in plasma ASGR1 levels compared with the non‐CAD group.

### 3.3 Association of ASGR1 With Cardiac Remodeling, Dysfunction, and Inflammatory Cytokines in MI Mice

Given that ASGR1 levels were highest in the MI group, we subsequently established a MI mouse model to further investigate its potential role in cardiac remodeling. As shown in Figure S1A,B, MI resulted in a marked increase in both collagen deposition and cardiomyocyte size compared with the Sham group. The percentage collagen area in the MI group was 21.53‐fold that in the Sham group (*p* = 0.001; Figure S1A), while the cross‐sectional area of cardiomyocytes was 1.77‐fold greater (*p* = 0.002; Figure S1B). Additionally, echocardiographic assessment (Figure S1C–G) revealed that MI mice exhibited increased LVEDd (1.35‐fold vs. Sham; *p* < 0.001; Figure S1D) and LVEDs (1.82‐fold vs. Sham; *p* < 0.001; Figure S1E) indicating significant left ventricular dilation. Conversely, LVEF% (Figure S1F) and FS% (Figure S1G) were markedly reduced in the MI group, reaching only 41.5% and 59.9% of those in the Sham group, respectively (both *p* < 0.001), confirming impaired left ventricular systolic function following MI. Moreover, compared with the Sham group, the MI group showed an approximately 3.03‐fold increase in *ASGR1* mRNA expression (*p* < 0.001; Figure S1H), 5.30‐fold and 11.23‐fold elevations in IL‐1β and IL‐6 levels (*p* = 0.002 and *p* < 0.001, respectively, Figure S1I,J), and a reduced IL‐10 level, which was only 51.0% of that in the Sham controls (*p* = 0.005; Figure S1K). Furthermore, Spearman correlation analysis further demonstrated strong positive correlations between ASGR1 expression and IL‐1β, IL‐6, CVF%, LVEDd, and LVEDs, while a negative correlation was observed between ASGR1 and FS% as well as LVEF% (Figure S1L).

### 3.4 Comparative Performance of ASGR1 and Conventional Biomarkers for Detecting Hypertensive CAD Patients

**FIGURE 2:**
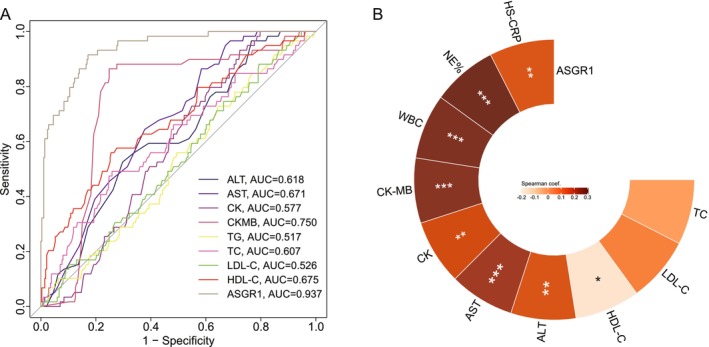
Comparative performances of ASGR1 and conventional biomarkers for detecting hypertensive CAD patients. (A) Comparison of ROC curves for distinguishing CAD from non‐CAD patients with hypertension using ASGR1, lipid profiles, CK, CK‐MB, ALT and AST, respectively. (B) Spearman rank correlation analyze was performed to assess the relationships between ASGR1 expression and inflammatory factors (hs‐CRP, NE% and WBC), liver injury biomarkers (AST and ALT), cardiac injury biomarkers (CK, CK‐MB) and lipid profiles (TC, LDL‐C and HDL‐C). ASGR1, asialoglycoprotein receptor 1; ROC, receiver operating characteristic; CAD, coronary artery disease; CK, creatine kinase; CK‐MB, creatine kinase MB; ALT, alanine aminotransferase; AST, aspartate aminotransferase; TG, triglyceride; TC, total cholesterol; HDL‐C, high‐density lipoprotein cholesterol; LDL‐C, low‐density lipoprotein cholesterol; hs‐CRP, high‐sensitivity C‐reactive protein; NE%, neutrophilic granulocyte %; WBC, white blood count. ***, *p* < 0.001; **, *p* < 0.01; *, *p* < 0.05.

In hypertensive patients, plasma ASGR1 exhibited superior diagnostic efficacy for hypertensive CAD patients compared with traditional biomarkers (Figure 2A). ROC curve analysis revealed ASGR1 had the highest AUC (0.937, 95% CI: 0.906–0.960), which significantly outperformed the following traditional biomarkers: CK (0.577, 95% CI: 0.523–0.630, *p* < 0.001), CK‐MB (0.750, 95% CI: 0.701–0.795, *p* < 0.001), ALT (0.618, 95% CI: 0.564–0.669, *p* < 0.001), AST (0.671, 95% CI: 0.619–0.720, *p* < 0.001), TC (0.607, 95% CI: 0.554–0.659, *p* < 0.001), TG (0.517, 95% CI: 0.463–0.571, *p* < 0.001), LDL‐C (0.526, 95% CI: 0.471–0.579), and HDL‐C (0.675, 95% CI: 0.623–0.725, *p* < 0.001). (Figure S1A). Meanwhile, inflammatory factors (hs‐CRP, NE% and WBC), liver injury biomarkers (AST and ALT), and cardiac injury biomarkers (CK, CK‐MB) were positively correlated with ASGR1, whereas HDL‐C was negatively correlated (Figure 2B).

### 3.5 Plasma ASGR1 Increased the Risk of CAD in Hypertensive Patients With Univariate and Multivariate Analyzes

As shown in Table S2, a total of 15 variables were identified as univariate correlates of CAD in the hypertensive cohort, encompassing behavioral (smoking status), biochemical (TC, HDL‐C, ALT, AST, CK, CK‐MB, hs‐CRP, WBC, NE%, and ASGR1), and echocardiographic (IVST, LVPW, LVEF, and LVM). In addition, after adjusting for the aforementioned confounding factors, each 1‐unit increase in plasma ASGR1 level was positively associated with a 37% higher odds of CAD (OR 1.37, 95% CI: 1.24–1.52, *p* < 0.001).

### 3.6 Association of ASGR1 and All‐Cause Rehospitalization

**FIGURE 3:**
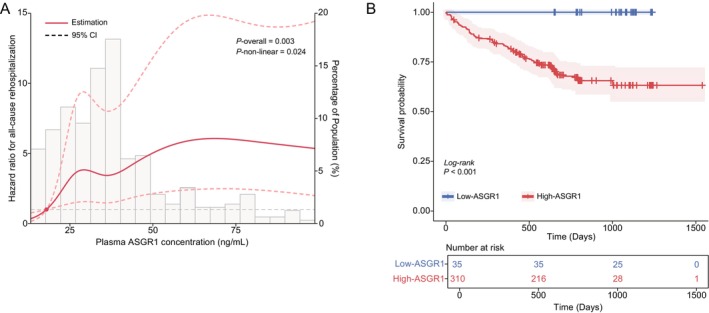
Association between plasma ASGR1 expression and rehospitalization during follow‐up. (A) The HRs (red lines) and 95% confidence intervals (dashed lines) were calculated based on the restricted cubic spline for the relationship between plasma ASGR1 concentrations and the risk of rehospitalization. (B) Kaplan–Meier curve for rehospitalization. Low‐ASGR1: ASGR1 ≤ 17.945 ng/mL (blue line); High‐ASGR1: ASGR1 > 17.945 ng/mL (red line). HR, hazards ratio; ASGR1, asialoglycoprotein receptor 1.

Furthermore, in the cohort study, we further investigated the association between plasma ASGR1 and all‐cause rehospitalization. RCS analysis revealed a correlation between plasma *ASGR1* and all‐cause rehospitalization (*p* overall = 0.003), and a non‐linear relationship (*p* non‐linear = 0.024) (Figure 3A). The Kaplan–Meier curve demonstrated the low‐ASGR1 group had a significantly higher probability of being free from all‐cause hospitalization compared to the high‐ASGR1 group (Log‐rank *p* < 0.001; Figure 3B). Additionally, univariate Cox regression analysis (Table 2) showed that a higher baseline ASGR1 level was associated with an increased risk of all‐cause hospitalization relative to a lower baseline level (HR: 2.37, 95% CI: 1.41–3.98, *p* = 0.001). Importantly, the result remained statistically significant after adjustment for potential risk factors that were identified as significant (*p* < 0.05) in the univariate analyses (adjusted HR: 1.97, 95% CI: 1.16–3.35, *p* = 0.012).

**TABLE 2:** Univariate and multivariate Cox analysis for the prediction of the all‐cause hospital readmission.

	HR (95% CI)	*P*	HR (95% CI)	*p*
Age (year)	0.99 (0.97–1.02)	0.505		
Sex, male	0.95 (0.62–1.46)	0.812		
BMI (kg/m^2^)	0.98 (0.92–1.04)	0.519		
SBP (mmHg)	1.00 (0.99–1.02)	0.578		
DBP (mmHg)	1.01 (0.99–1.03)	0.401		
Antihypertensive medication	1.05 (0.63–1.75)	0.861		
Smoking	1.26 (0.83–1.91)	0.287		
Drinking	1.07 (0.68–1.67)	0.783		
T2DM	1.74 (1.15–2.64)	0.009	1.61 (1.01–2.58)	0.047
FBG (mmol/L)	1.12 (1.05–1.19)	< 0.001		
TG (mmol/L)	1.01 (0.82–1.24)	0.943		
TC (mmol/L)	0.98 (0.81–1.18)	0.792		
HDLC (mmol/L)	0.72 (0.39–1.33)	0.298		
LDLC (mmol/L)	1.09 (0.89–1.33)	0.427		
ALT, U/L	1.12 (0.94–1.33)	0.196		
AST, U/L	1.17 (1.02–1.36)	0.030		
CK, U/L	1.19 (1.02–1.39)	0.029		
CKMB, ng/mL	1.25 (1.06–1.48)	0.009		
Hs‐CRP, mg/L	1.06 (0.89–1.26)	0.499		
WBC, ×10^9^/L	1.12 (1.06–1.18)	< 0.001		
NE%	1.03 (1.01–1.05)	0.001		
LVEDd, cm	1.18 (0.83–1.67)	0.352		
LVEDs, cm	1.14 (0.81–1.59)	0.462		
IVST, cm	1.48 (1.27–1.72)	< 0.001		
LVPW, cm	1.58 (1.26–1.97)	< 0.001		
LAD, cm	1.39 (0.93–2.06)	0.106		
LVEF, %	0.97 (0.95–0.99)	0.002		
LVM, g	1.43 (1.21–1.70)	< 0.001		
Higher ASGR1	2.37 (1.41–3.98)	0.001	1.97 (1.16–3.35)	0.012

Abbreviations: CI, confidence interval; HR, hazard ratio. Other abbreviations are shown in Table 1.

## 4 Discussion

In the present study, we first demonstrated that plasma ASGR1 levels were significantly higher in hypertensive patients with CAD than in those with hypertension alone. Notably, higher ASGR1 expression is accompanied by severe coronary lesions. Specifically, plasma ASGR1 levels were markedly higher in patients with NSTEMI and STEMI than in those with UA. Furthermore, we established an in vivo mouse model of MI, in which we confirmed elevated ASGR1 levels post‐MI. We also found a significant positive correlation between ASGR1 and cardiac remodeling, cardiac dysfunction, as well as proinflammatory cytokines. Besides, in a further follow‐up study, we found that an elevated baseline level of ASGR1 was also associated with an increased risk of all‐cause rehospitalization.

Hypertension is a well‐established major risk factor for CAD, with complex and multifactorial pathophysiology involving neurohormonal overactivation, endothelial dysfunction, accelerated atherosclerosis, impaired coronary microcirculation, hypertension‐mediated cardiovascular damage, and increased arterial stiffness that compromises coronary perfusion. Given the close interplay between hypertension and CAD, identifying key molecules linking these conditions holds important clinical significance [32]. In the present study, patients with hypertension comorbid CAD exhibited significantly worse biochemical and echocardiographic profiles than those with hypertension alone, indicating a more advanced pathological phenotype characterized by aggravated myocardial injury, systemic inflammation, left ventricular hypertrophy, and adverse myocardial remodeling. Elevated liver enzymes (ALT, AST) further suggested concurrent hepatic congestion or metabolic disturbances (Table 1). Collectively, these abnormalities represent established risk factors for cardiac dysfunction and predict unfavorable long‐term prognosis in this high‐risk population, which is in line with our previous study [20].

Since 2016, ASGR1 has gained considerable attention from researchers, largely due to its key role in regulating cholesterol metabolism [11] and can affect plasma cholesterol levels by modulating the clearance of LDL‐C [33]. Loss‐of‐function variants in *ASGR1*, such as the *del12* mutation, are associated with reduced plasma non‐HDL cholesterol levels and a 34% lower risk of CAD [11]. Moreover, under Western diet feeding, ASGR1 deficiency in *Asgr1* ^*−/−*^ *ApoE* ^*−/−*^ mice attenuated atherosclerotic lesion formation, whereas ASGR1 overexpression exacerbated atherosclerosis, suggesting that targeting ASGR1 may serve as a novel therapeutic strategy for atherosclerosis and cardiovascular diseases [14]. Intriguingly, a prior study has revealed that the impact of ASGR1 on LDL‐C levels differs between hypertensive and non‐hypertensive patients. Specifically, in hypertensive patients, ASGR1 was positively correlated with LDL‐C levels, whereas this relationship could not be observed in non‐hypertensive patients [34]. It is well established that LDL‐C is a crucial risk factor for atherosclerosis, which serves as the core pathological basis of CAD [35]. However, the relationship between ASGR1 expression levels and CAD, particularly in terms of the severity of coronary artery lesions and plaque stability in hypertensive patients, remains poorly understood.

In the present study, we observed that ASGR1 expression was elevated in CAD patients. Notably, this elevation was more pronounced in those with multi‐vessel lesions (CAG > 2) and in ACS patients, particularly among MI cases, including NSTEMI and STEMI (Figure 1). Previous research has shown that ASGR1 expression is markedly higher in patients with multi‐vessel CAD (involving more than 2 vessels) and those with AMI when compared to non‐CAD patients [15]. To a certain extent, these findings support the results of our present study, although our research specifically focuses on hypertensive patients.

Mechanistically, ASGR1 exerts multifaceted biological effects, with its roles in lipid metabolism and inflammation being the most well‐characterized. On the one hand, previous study demonstrated that ASGR1 deficiency decreases lipid levels by promoting cholesterol transport to high‐density lipoprotein and cholesterol excretion to bile and feces [33]. Consistent with previous study, we observed a negative correlation between ASGR1 and HDL‐C, along with a positive correlation between ASGR1 and both LDL‐C and TC, albeit the latter associations did not reach statistical significance (Figure 2). On the other hand, ASGR1 is also intricately involved in the inflammatory process [17]. Early evidence from Luo et al. proposed that ASGR1 expression levels are correlated with hs‐CRP and WBC counts, both of which are well‐established indicators of systemic inflammation [15]. In line with these existing findings, our study has further uncovered that ASGR1 was positively correlated with pro‐inflammatory cytokines, such as IL‐1β, IL‐6, hs‐CRP and WBC counts but negatively associated with IL‐10 (Figure S1). Prior study indicated that ASGR1‐knockdown mice suppressed inflammatory monocytes in peripheral blood mononuclear cells (PBMCs) and also decreased the level of IL‐1β, IL‐6, TNF‐α and alleviated liver injury [36]. Moreover, Ye et al. found that a causal association between ASGR1 inhibitor and heart failure (HF), suggesting genetic evidence for the anti‐inflammatory role of ASGR1 inhibitors in reducing HF risk using Mendelian randomization analysis. Above all, we speculated that ASGR1 regulates hypertension complicated with CAD through modulation of lipid metabolism and inflammatory responses, though the specific underlying mechanisms require further investigation in subsequent studies. In terms of clinical value, the diagnostic efficacy of ASGR1 is superior to traditional myocardial injury markers (e.g., CK, CK‐MB), suggesting that it may serve as an early screening indicator for hypertensive patients with concomitant CAD. Meanwhile, longitudinal follow‐up data indicated that elevated ASGR1 expression was linked to a higher risk of CAD progression a significantly increased all‐cause readmission rate (Table 2). In line with this, previous studies have found increased levels ASGR1 was associated with CVD or mortality [37, 38].

Nonetheless, our study has several limitations. Firstly, as a single‐center observational study with a limited sample size, our findings lack sufficient generalizability. Therefore, larger multicenter cohorts are needed to validate the results. Secondly, the status of ASGR1 was obtained only once at baseline; tracking the dynamics of plasma ASGR1 and all‐cause readmission in the longitudinal study might provide more robust evidence. Thirdly, all observed associations are non‐causal, as we cannot determine whether elevated ASGR1 contributes directly to adverse outcomes or merely reflects myocardial injury such as myocyte necrosis. In addition, the lack of quantified infarct size data restricts adjustment for this important confounder related to survival and recurrent events. Our longitudinal link between higher baseline ASGR1 and increased all‐cause rehospitalization should be interpreted as an observational association only. Future studies with mechanistic analyses and quantified infarct size are warranted to clarify these relationships. Lastly, although ASGR1 was associated with lipid and inflammatory markers, the specific mechanisms underlying its role in CAD development and progression remain to be verified by gene overexpression and knockout experiments to establish its causal role in post‐MI remodeling. At the cellular level, gain‐ and loss‐of‐function studies in cardiomyocytes and cardiac fibroblasts are needed to dissect the cell‐autonomous mechanisms by which ASGR1 modulates inflammation and fibrosis using Western blot and immunofluorescence and other related assays to verify the present observations. Elucidating the downstream signaling pathways and ligand interactions of ASGR1 in this context will be critical for understanding its therapeutic potential.

## 5 Conclusion

This study provides the first evidence that ASGR1 is significantly upregulated in hypertensive CAD patients and correlated with disease severity and adverse outcomes. Meanwhile, we also found that ASGR1 has a significant correlation with lipids and inflammatory factors. Our findings suggest that ASGR1 has the potential to serve as a diagnostic biomarker, offering new perspectives for CAD management. Further investigations are warranted to elucidate its molecular mechanisms and clinical applications.

## Author Contributions

Conceptualization: Lin Zhao, Jiuchang Zhong, and Ying Dong. Methodology: Ying Liu, Xinyu Zhou, Peng Hao, Kuibao Li, and Jing Li. Validation: Ying Guo, Ning Wang, Hongjie Chi, Jing Li, Hong Li, and Xiaoyan Yang. Formal analysis: Ying Liu and Xinyu Zhou. Writing – original draft preparation: Ying Liu and Xinyu Zhou. Writing – review and editing: Lin Zhao, Jiuchang Zhong, and Ying Dong. Supervision: Lin Zhao, Jiuchang Zhong, and Ying Dong.

## Funding

The authors have nothing to report.

## Ethics Statement

The study was approved by the Ethics Committee of Beijing Chaoyang Hospital (No. 2022‐ke‐483). All patients involved in this research had complete clinical data. Written informed consent was obtained from all participants.

## Conflicts of Interest

The authors declare no conflicts of interest.

## Supporting information


**Figure S1:** Cardiac ASGR1 expression and its correlation with cardiac functional, histological alterations and inflammatory cytokines. (A‐B) Heart tissue cross‐sections were stained with Masson's trichrome and WGA, respectively, to evaluate cardiac fibrosis and cardiomyocyte cross‐sectional area. (C) Cardiac function was examined by echocariography, and representative images of M‐mode echocardiography are shown. (D‐G) Quantification of LVEDd, LVEDs, LVEF%, and FS%. (H) The mRNA expression of ASGR1 in cardiac tissue from Sham and MI mice was determined by quantitative real‐time PCR (qRT‐PCR). (I‐K) Cardiac levels of pro‐inflammatory cytokines IL‐1β (I) and IL‐6 (J), as well as the anti‐inflammatory cytokine IL‐10 (K), were determined. Data are presented as mean ± SEM (*n* = 5 per group). Statistical significance was determined using unpaired Student's *t* test. (L) Spearman rank correlation analyze was performed to assess the relationships between ASGR1 expression and cardiac functional, histological alterations and inflammatory cytokines. ASGR1, asialoglycoprotein receptor 1; MI, myocardial infarction; LVEDd, left ventricular end‐diastolic diameter; LVEDs, left ventricular end‐systolic diameter; LVEF%, left ventricular ejection fraction; FS%, fractional shortening; IL, interleukin; CAS‐CM, cardiomyocyte cross‐sectional area of cardiomyocytes; CVF%, collagen volume fraction; SEM, standard error of the mean. ***, *p* < 0.05.
**Table S1:** Primer sequences (mouse) for quantitative PCR.
**Table S2:** Univariate and multivariate logistic regression of CAD patients with hypertension.

## Data Availability

Privacy/ethical restrictions.
