# Supplementary material for: Elevated ASGR1 as a Potential Diagnostic Biomarker for Coronary Artery Disease and Predictor of Adverse Outcomes in Hypertensive Patients
Source: FASEB J. 2026 Apr 28;40:e71859. doi: 10.1096/fj.202504652RR (PMC13123747; doi:10.1096/fj.202504652RR)
Supplement: Supplementary file 1 — Figure S1: Cardiac ASGR1 expression and its correlation with cardiac functional, histological alterations and inflammatory cytokines. (A‐B) Heart tissue cross‐sections were stained with Masson's trichrome and WGA, respectively, to evaluate cardiac fibrosis and cardiomyocyte cross‐sectional area. (C) Cardiac function was examined by echocariography, and representative images of M‐mode echocardiography are shown. (D‐G) Quantification of LVEDd, LVEDs, LVEF%, and FS%. (H) The mRNA expression of ASGR1 in cardiac tissue from Sham and MI mice was determined by quantitative real‐time PCR (qRT‐PCR). (I‐K) Cardiac levels of pro‐inflammatory cytokines IL‐1β (I) and IL‐6 (J), as well as the anti‐inflammatory cytokine IL‐10 (K), were determined. Data are presented as mean ± SEM (n = 5 per group). Statistical significance was determined using unpaired Student's t test. (L) Spearman rank correlation analyze was performed to assess the relationships between ASGR1 expression and cardiac functional, histological alterations and inflammatory cytokines. ASGR1, asialoglycoprotein receptor 1; MI, myocardial infarction; LVEDd, left ventricular end‐diastolic diameter; LVEDs, left ventricular end‐systolic diameter; LVEF%, left ventricular ejection fraction; FS%, fractional shortening; IL, interleukin; CAS‐CM, cardiomyocyte cross‐sectional area of cardiomyocytes; CVF%, collagen volume fraction; SEM, standard error of the mean. ***, p < 0.05. Table S1: Primer sequences (mouse) for quantitative PCR. Table S2: Univariate and multivariate logistic regression of CAD patients with hypertension. [file FSB2-40-e71859-s001.pdf]

Supplementary material

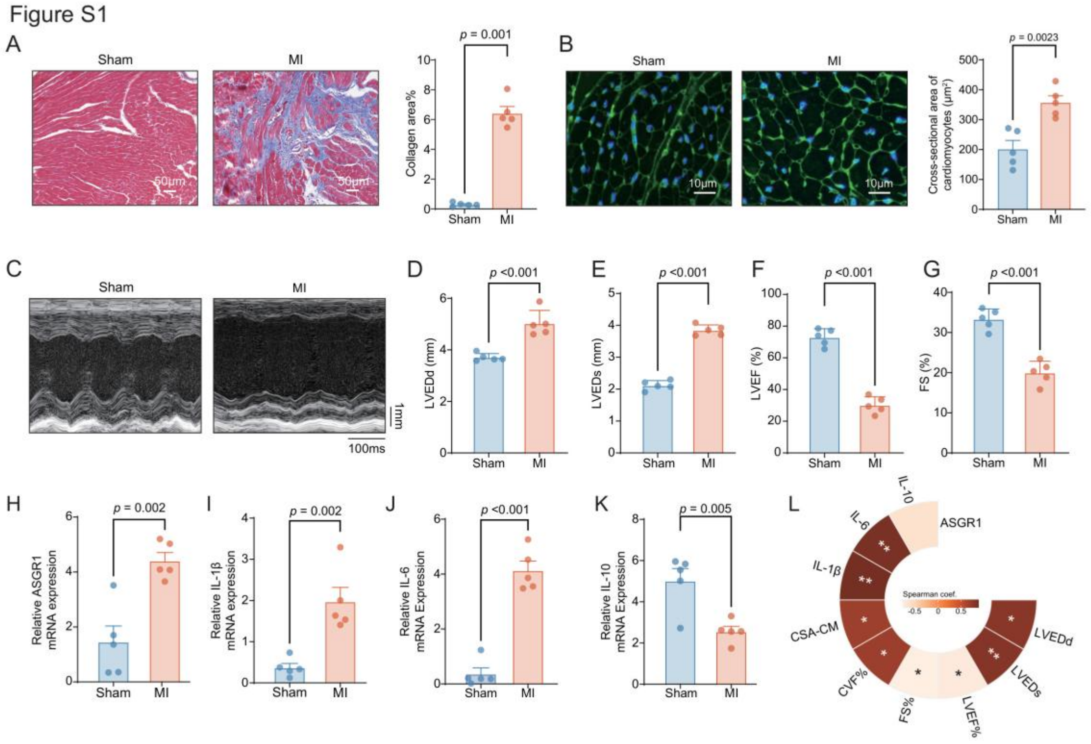

Figure S1. Figure S1. Cardiac ASGR1 expression and its correlation with cardiac functional, histological alterations and inflammatory cytokines. (A-B) Heart tissue cross-sections were stained with Masson's trichrome and WGA, respectively, to evaluate cardiac fibrosis and cardiomyocyte cross-sectional area. (C) Cardiac function was examined by echocariography, and representative images of M-mode echocardiography are shown. (D-G) Quantification of LVEDd, LVEDs, LVEF%, and FS%. (H) The mRNA expression of ASGR1 in cardiac tissue from Sham and MI mice was determined by quantitative real-time PCR (qRT-PCR). (I-K) Cardiac levels of pro-inflammatory cytokines IL-1β (I) and IL-6 (J), as well as the anti-inflammatory cytokine IL-10 (K), were determined. Data are presented as mean ± SEM (n=5 per group). Statistical significance was determined using unpaired Student's t-test. (L) Spearman rank correlation analyze was performed to assess the relationships between ASGR1 expression and cardiac functional, histological alterations and inflammatory cytokines. ASGR1, asialoglycoprotein receptor 1; MI, myocardial infarction; LVEDd, left ventricular end-diastolic diameter; LVEDs, left ventricular end-systolic diameter; LVEF%, left ventricular ejection fraction; FS%, fractional shortening; IL, interleukin; CAS-CM, cardiomyocyte cross-sectional area of cardiomyocytes; CVF%, collagen volume fraction; SEM, standard error of the mean. ***, p <0.001; **, p <0.01; *, p <0.05.

Table S1. Primer sequences (mouse) for quantitative PCR.

Genes	Forward primer (5'-3')	Reverse primer (5'-3')
ASGR1	CCTCCCACTCCACGGCTCTTG	ACACAGACAACCACCAGCAACAG
IL-1β	CAAATGCCACCTTTTGACAGTG	TGGATGCTCTCATCAGGACAG
IL-6	GGGACTGATGCTGGTGA	GGCTTTGTCTTTCTTGT
IL-10	TCCCTGGGTGAGAAGCTGAAGAC	CACCTGCTCCACTGCCTTGC
GAPDH	AGTGCCAGCCTCGTCTCATA	TGAACTTGCCGTGGGTAGAG

Abbreviations: PCR, polymerase chain reaction; ASGR1, asialoglycoprotein receptor 1; IL-1β: interleukin-1 beta; IL-6, interleukin-6; IL-10, interleukin-10; GAPDH, glyceraldehyde-3-phosphate dehydrogenase.

Table S2 Univariate and multivariate logistic regression of CAD patients with hypertension.

	OR (95% CI)	*P* value	OR (95% CI)	*P* value
Age (year)	0.98 (0.94-1.01)	0.152		
Sex, male	1.21 (0.67-2.20)	0.526		
BMI (kg/m^2)	0.96 (0.89-1.04)	0.295		
SBP (mmHg)	1.00 (0.98-1.01)	0.686		
DBP (mmHg)	1.01 (0.98-1.03)	0.707		
Antihypertensive medication	0.56 (0.25- 1.23)	0.148		
Smoking	2.52 (1.22-5.18)	0.012		
Drinking	1.26 (0.64-2.46)	0.502		
T2DM	0.83 (0.47-1.46)	0.526		
FBG (mmol/L)	1.03 (0.93-1.15)	0.581		
TG (mmol/L)	0.97 (0.72-1.30)	0.820		
TC (mmol/L)	0.70 (0.54-0.91)	0.007		
HDL-C (mmol/L)	0.17 (0.07-0.39)	<0.001	0.12 (0.02-0.68)	0.017
LDL-C (mmol/L)	0.94 (0.70-1.24)	0.640		
ALT, U/L	1.04 (1.01-1.07)	0.004		
AST, U/L	1.05 (1.02-1.09)	0.003		
CK, U/L	1.00 (1.00-1.01)	0.017		
CKMB, ng/mL	1.01 (1.00-1.01)	<0.001		
Hs-CRP, mg/L	1.09 (1.01-1.18)	0.023		
WBC, ×10^9/L	1.32 (1.15-1.50)	<0.001		
NE%	1.05 (1.03-1.08)	<0.001		
LVEDd, cm	0.77 (0.46-1.29)	0.321		
LVEDs, cm	0.79 (0.49-1.26)	0.322		
IVST, cm	4.37 (2.73-6.99)	<0.001	4.32 (1.06-17.67)	0.042
LVPW, cm	1.97 (1.45-2.67)	<0.001		
LAD, cm	1.44 (0.89-2.35)	0.138		
LVEF, %	0.95 (0.92-0.99)	0.010		
LVM, g	1.02 (1.01-1.02)	<0.001		
ASGR1	1.30 (1.21-1.39)	<0.001	1.37 (1.24-1.52)	<0.001

Abbreviations: OR, odds ratio; CI, confidence interval. Other abbreviations are shown in Table 1.
